
JOURNAL INFORMATION
==============================
NLM Title Abbreviation: Atlanta Med Surg J (1884)
NLM Journal ID: 101251623

Atlanta Medical and Surgical Journal (1884)

Publisher: Biomedical Journal Digitization Project

ARTICLE INFORMATION
==============================
PMCID: PMC9201862
PMID: 35829610
Article version: 1
Subjects: Editorial Notes and Comments

So-called Christian Science

Print publication date: 1899 Feb
Volume: 15
Volume Series: New Ser
Issue: 12
First page: 825
Last page: 832
License: This work is free of known copyright restrictions. For more information, please see PMC Back Issue Digitization.
License URL: https://creativecommons.org/publicdomain/mark/1.0/

==============================

### SO-CALLED CHRISTIAN SCIENCE.

SINCE the untimely death of Harold Frederic, in England, who was at that time under the medical care of a woman Christian Scientist, the medical journals have been teeming with paragraphs condemning the practices of this sect of people. In the case of Frederic, it was ascertained from a court of inquiry that his life might have been saved, or at least much prolonged, had he received rational scientific treatment from a reputable physician. The English papers especially are very severe in their criticisms, and this seems to be the general sentiment also on the continent. The *St. James' Gazette*, published in London, is very pronounced in its ideas. It says: "Apart altogether from this individual case, which has resulted in the loss of a life of much promise, we trust that the prominence now given to the methods of the enterprising American ladies who are making a fat living out of the weak-minded and credulous, will result in their receiving systematic treatment at the hands of the criminal investigation department and the director of public prosecutions. The vulgar quack, or vender of 'charms,' is watched and moved on by the police, and there is no reason why these people, simply because they add blasphemy and pseudo-science to the old-fashioned procedure, should flourish unchecked. In France or Germany they would find themselves in prison or across the frontier in a week, and we trust there is some equally effective weapon in the hands of our own police."

This is severe, but to all intelligent thinking people it is deserved.

The *Standard*, the *Daily News*, the *Morning Post*, *Graphic*, and *Leader*, all prominent English papers, demand "summary punishment for the people who caused Harold Frederic's death," and some few of the editorials contain remarks which are anything but flattering to this country, from which this "pagan superstition" is supposed to have been imported.

The *Medical News* informs us that "for the second time within two weeks, a Cincinnati, Ohio, jury convicted, and a Cincinnati judge has fined, a practitioner of 'Christian Science,' who had been arrested on the charge of violating the State law which forbids any but duly licensed doctors to treat the sick for hire."

Since the time of Frederic's death, more than half a dozen other deaths have been reported, which, in all human probability, stood an excellent chance for recovery under the appropriate care of a physician.

Even in Atlanta, the death of one man, who would not call in a regular physician, but continued under the administration of one of the attachés of the Christian Science sect, brought forth loud cries of indignation from the neighbors around. Surely it is time for physicians to take a prominent stand against such iniquitous and uncivilized proceedings! But there is another aspect of the subject which we wish to speak of, and which, in itself, is a partial guarantee for the safety of the public: The Atlanta Christian Science church has grown so large that their present quarters are inadequate, so that they are now erecting a temple to meet these demands. Atlanta, as the Mecca of Christian Science, is to the South what Boston is to the North. In the church, as we understand it, there are a number of "administers," who are delegated with the power of healing physical suffering, and these "administer," either in the church or at the homes of the patient. This administration is not entirely gratuitous, for it is one of the ways for raising funds for the sustenance of the church, but more especially for the "administrator."

The charges, as we hear, are one dollar for each "administration," and some of them even have offices, just as regular practicing physicians. Now this is totally in violation of the State law governing the practicing physician, which clearly says that such "must be a graduate of a regularly incorporated medical college in good standing, and must have stood an examination before the State Medical Examining Board, and have received a license from such before they can practice in the State." This, of course, is applicable only to those who receive fees for their services.

The Christian Scientist charges one dollar for his or her, usually her, "administration," and in doing so violates the laws of the State, and is punishable with fine.

Still this practice goes on right in the city of Atlanta, and the State authorities have not interfered. This should be brought to their attention by the State Medical Examining Board, and, as in Cincinnati, steps taken for its correction.

"A word to the wise is sufficient."

---

THE ATLANTA MEDICAL AND SURGICAL JOURNAL has now, more than ever, entered the field of progressive activity. We refer to its past achievements, in giving to the profession one of the best and cleanest medical periodicals in the South, with a feeling of laudable pride. Our pages have never contained anything which the most ethical physician could not commend. Such will be our policy in the future. We are a guarantee for every advertisement found in the pages of THE JOURNAL, for we have taken none for whom we cannot make this statement with the greatest feeling of security. Our readers, therefore, need never doubt the ethical character of the contents of this JOURNAL. In addition to this, we wish to announce that the yearly subscription price has been reduced to one dollar. This does not mean a cheapening in the character of the contents of THE JOURNAL, but it means that we wish to have more readers, who, perhaps, are not able to pay the former subscription price during these hard times. The size of THE JOURNAL has nowhere been reduced, and when we say this, it means that our expenses are the same as before, and yet we are going to give to the profession this same journal for just one-half the price as previously. At the first meeting of the Atlanta Society of Medicine, in January, a resolution was unanimously adopted making this JOURNAL the official organ of that society. This means that all the papers read and all the discussions and proceedings of that body will be published by us. This society seems now in the beginning of a wave of prosperity, and its proceedings will be very instructive and interesting to all the readers of THE JOURNAL. Discussions of any medical subject are always instructive. We will also print *alumni notes* in regard to the College of Physicians and Surgeons, and the old and new graduates will always find a medium through which and from which information can be conveyed.

---

SEVERAL weeks ago the daily newspapers were filled with notices concerning a remarkable operation of restoring vision to a blind person bythe implantation of a piece of the cornea of a rabbit into that of the patient. This operation was performed by Dr. C. H. Kohler, of Minneapolis, Minn. While reading these garbled newspaper reports, we knew that the roseate results depicted were not likely to be realized. Like the paper read by Dr. Murphy, of Chicago, on a new cure for consumption, we did not wish to pass judgment on it before seeing the original paper. We knew of the various trials which have been made by ophthalmologists all over the world to accomplish just that one thing which the daily papers stated had been accomplished by Dr. Kohler. We have seen it tried by such eminent men as Fuchs, of Vienna, and Von Hippel, of Halle, the latter having originated the trephine which is now used in attempting the operation, and by both without success. We have just read Dr. Kohler's original article on the operation, published for the first time in the *Medical Dial* of Minneapolis, and like the true physician that he is, he deplores the newspaper notoriety that he has obtained, and gives the real merits of the operation. The doctor, however, is to be congratulated on even the amount of success obtained, as it is more than others have obtained. The trouble has been that the operation is usually successful for a few weeks, but this condition does not last. We trust that in Dr. Kohler's case the results thus far obtained may be lasting, yet he closes his article with this sentence: "As it now appears, the question of sight is decidedly an uncertain quantity."

---

THE ATLANTA SOCIETY OF MEDICINE has organized for the year 1899 with renewed vigor. The officers for this year were given in the last issue, and these names in themselves insure an active organization. A new feature of the society, contemplated for some time, but just now begun to be put into active operation, is the organization of a medical library for the use of the members and visiting physicians who reside outside of the city. A recent law passed by the society requires one-half of the collected dues for each year to be placed to the fund used in increasing the completeness of the library, and this, besides voluntary subscriptions, will certainly form a most excellent nucleus for the formation of a medical library. The society now has a large, beautiful room in which to meet, situated on the tenth floor of the English-American building, and the meetings for this year promise to be most excellent. We take the liberty here to ask all medical journals who will to kindly send us, gratuitously, a copy of their journal to be placed on the table in the library, and we also ask the publishing houses and authors of medical works to aid us with complimentary copies of their works. Such can be sent to the librarian, Dr. Michael Hoke, English-American building, Atlanta, Ga., and a full appreciation of the same will be acknowledged.

---

THERE should be some law to prevent plagiarism, we mean especially in medical journalism. Every day we see medical journals who start off their "Original Articles" with an article by a prominent physician or surgeon, and when you have read it through, there is absolutely no mark which would indicate it to be more than an article "written expressly" for that journal. To the majority of readers such is the impression, yet the editor or physician who looks over a large number of journals will readily recognize it as taken from some other medical periodical. There would be some extenuation for such decrepids did they "give honor to whom honor was due," but instead of that they wish to delude their readers by placing such an article in a prominent place and calling it original. Journals which are so puny that medical men will not furnish them articles from their own pen, "should fold their tent and silently steal away," or they should organize a mutual aid society for the dissemination of the virtues found in Ayer's Perry Chectoral.

---

THE *Tri-State Medical Journal and Practitioner* in its Christmas garb of colored halos reminds one more of a weekly, secular, illustrated periodical than the somber pages of a medical journal. Nor are we far wrong if the interior pages are examined. Besides having a number of excellent medical articles, it also contains two very pleasing little sketches which would prove entertaining to any layman. The first entitled "Faithful Unto Death," is from the pen of Dr. G. Frank Lydston, of Chicago, and like some of his other sketches, it is written in the doctor's happiest vein. The other is called "Dr. Jim's Atlantean Tumor," by Dr. W. B. Outten, and is also an amusing sketch. We are glad to see a physician's literary ability manifested in other ways than in the production of medical brochures, and the writings of Dr. Weir Mitchell, and lately of Sir Grainger Stewart, certainly stamp the medical profession as containing literary lights among its membership.

---

In the death of Dr. John B. Hamilton, of Chicago, Ill., the medical profession of the United States has lost one of its most valued members. Dr. Hamilton had unusual qualities of executive ability, and coupled with this was the possession of a mind filled with extensive learning. At the late Quarantine Convention held in Memphis, Tenn., his words and advice were listened to with marks of exceptional interest, because on this one subject especially was he an authority well versed in every detail. It was perhaps as editor of *The Journal of the American Medical Association* that his signal ability was most marked, both in its editorial columns and on the floor of the Association's annual meetings. It has been largely through his own individual work that this journal has achieved such phenomenal success. Personally, Dr. Hamilton was was one of the most agreeable and magnetic men that it has ever been our pleasure to meet. We deeply regret his untimely death at the very noontide of his activity.

---

We can certainly unite heartily with the *Pennsylvania Medical Journal* in paying a parting tribute to the *Philadelphia Polyclinic*. This little journal was always a welcome visitor on our exchange table because its contents were always clean, interesting and thoroughly scientific. Dr. Cohen, as editor, is certainly to be congratulated on the favorable comments which have been paid this little periodical. In the future its interests will be allied with those of the *Philadelphia Medical Journal,* and we have no doubt of the substantial aid the latter will receive.

---

THE first number of a new medical journal is before us. It is *The St. Paul Medical Journal,* published monthly in St. Paul, Minn. It has a staff of excellent assistant editors and collaborators of wide prominence. We do not know when we have received a medical journal whose general appearance was so pleasing. We welcome it among our list of exchanges and will look forward with pleasure and profit to its monthly reception.

---

THE *North Carolina Medical Journal* has begun the twenty-second year of its existence under new management. The new editors and proprietors are Drs. Robert L. Gibbons, Robert D. Jewett and W. H. Wakefield. In the future it will be published in Charlotte, N. C. The first issue under the new management is excellent, and we extend our best wishes to the editors.

---

WE note with pleasure the following order in reference to Dr. Grandy, our former colleague in the editorial department of THE JOURNAL:

HEADQUARTERS U. S. FORCES,
CAMP ONWARD,
SAVANNAH, GA., January 6, 1899.

GENERAL ORDER
No. 15.

The following appointment is hereby announced:

*Luther B. Grandy,* Major 3d Georgia Volunteer Infantry, to be Chief Surgeon.

By command of Col. Berner.

J. M. KIMBROUGH, JR.,
1st Lieut. and Assistant Adjutant-General.

OFFICIAL:
